# Facilitators and barriers to effective primary health care in Zimbabwe

**DOI:** 10.4102/phcfm.v9i1.1639

**Published:** 2017-11-22

**Authors:** Sunanda C. Ray, Nyasha Masuka

**Affiliations:** 1 Department of Community Medicine, College of Health Sciences, University of Zimbabwe, Zimbabwe; 2 Ministry of Health and Child Care, Matabeleland North, Zimbabwe

## Overview of country and burden of disease

Following impressive health gains in the first decade following independence in 1980, the health sector in Zimbabwe has been severely undermined by the human immunodeficiency virus (HIV) epidemic and economic crises, which worsened from the mid-1990s. Two years after the Alma Ata Declaration in 1978, the government adopted the Primary Health Care (PHC) approach, directing resources towards disadvantaged areas and active participation of communities in transforming their health.^1^ National health strategies have continued to endorse the PHC approach as underpinning health provision,^2^ but skills migration, low investment and limited resources have deterred this ambition. The near collapse of the health system in 2008 was followed in 2009–2012 by some recovery of the economy and renewed investment in health services.^2^ Economic growth declined again during 2013–2017, with 72% of the population now living below the national poverty line and 21% living on less than $1.90 a day.^3^ The maternal mortality ratio remains high at 651 deaths per 100 000 live births; the under-5 child mortality rate is 69 deaths per 1000 live births and 27% of children under 5 are stunted.^4^ Human immunodeficiency virus prevalence rose from 15% in adults aged 15–49 years in 1990, peaked at 22% in 2000, and is now 14%, 17% in women and 11% in men.^4^ Life expectancy at birth went from 58 years in 1990 to 45 years in 2000 and 60 years in 2016, following introduction of anti-retroviral therapy for HIV.^3^ Tuberculosis, malaria and water-borne diseases continue to be high in prevalence, while non-communicable diseases are now 31% of the disease burden.^5^

## Current Primary Health Care system

The population of Zimbabwe is 16 million (67% rural and 33% urban).^3^ Table 1 shows the number of hospitals and PHC facilities that serve this population. The public PHC workforce is largely nurse-led, with PHC nurses in rural clinics and nurses, midwives and clinical officers in urban municipality clinics, hospital outpatients and inpatients. Nurse-anaesthetists provide the majority of anaesthesia in urban and rural hospitals, where caesarean sections are the main surgical procedure. Doctors in public PHC provide supervision and teaching, develop guidelines and consult on referred cases. Nearly every district (±250 000 population) has at least two medical officers; every PHC centre has at least two qualified nurses; 59% of administrative wards have an environmental health technician and 60% of villages have access to a village health worker.^2^

**TABLE 1:** Health facilities profile in Zimbabwe.

Hospital facilities	*n*	Primary health facilities	*n*
Central hospitals	6	Clinics	1122
Provincial hospitals	8	Polyclinics	15
District hospitals	44	Mission clinics	25
Mission hospitals	62	City council/municipal clinics	96
Rural hospitals	62	Rural health centres	307
-	-	Private clinics	69
**Total**	**214**	**-**	**1634**

*Source*: National Health Strategy.^2^

## Weaknesses

Financial barriers and user fees are major barriers to care, with 7.6% of households incurring catastrophic health expenditure in 2015, 13% in the poorest and 3% in the richest households.^6^ Total health expenditure in 2015 was $103.80 per capita, equivalent to 10.3% of nominal gross domestic product. Only 21% of health financing is from government, while households contribute 25% through out-of-pocket expenditure plus private contributions to health insurance, corporations and employers (28%) and donors (25%). Private insurance schemes are doctor-led and urban-based, serving less than 10% of the population. Unemployment is high with most people now in informal employment with no benefits. Curative care takes 65% of government health expenditure (mainly salaries), while prevention only receives 24% and administration 10%. Government funding for health constitutes 8.7% of total government expenditure.^6^

## Strengths

**FIGURE 1:**
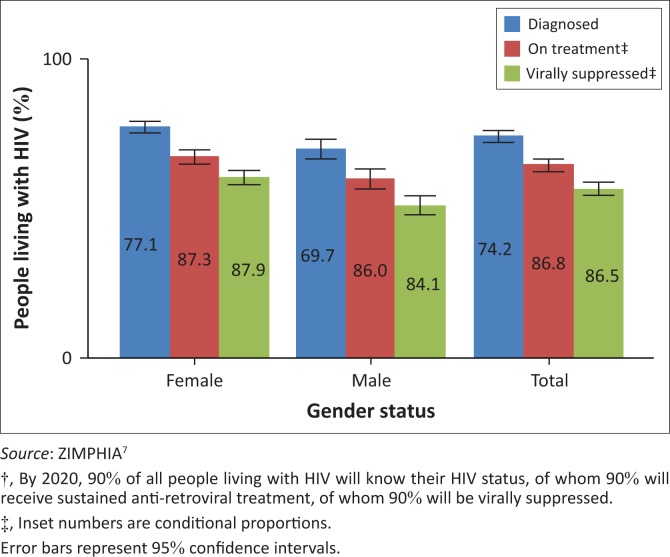
Achievements of the HIV programme against the 90:90:90 goals†.

Funding from donors increased from $167 million in 2009 to $361 million in 2015.^2,4^ External funding has been essential for health system strengthening, retention of health workers, procurement and distribution of commodities. Donor funding of HIV programmes has led to impressive uptake of anti-retroviral treatment (Figure 1) and prevention of mother-to-child transmission^7^, although such vertical approaches may undermine local ownership, innovation and leadership. Quality improvement initiatives led by the Ministry of Health, albeit top-down, have the potential for district-level impact when accompanied by mentorship and training. Use of root cause analysis in identifying factors contributing to maternal and perinatal deaths has demonstrated reduced deaths in one province. Similarly, utilisation of data for local decision-making has resulted in greater ownership and innovation at district and provincial levels. Mentorship visits from specialists in obstetrics, anaesthetics and paediatrics to provincial and district hospitals have led to improved morale and better accountability amongst medical officers.

## Current place of family medicine in the health system

Postgraduate specialist training (Master’s in Medicine, MMed) in family medicine is going through approval for the University of Zimbabwe, Harare, and National University of Science and Technology, Bulawayo. First intakes for training are anticipated at both universities for 2018. The four-year programme will be based at district and provincial hospitals, close to populations of greatest need. The programme’s success relies on having provincial hospital specialists in obstetrics, anaesthetics, paediatrics, general surgery and internal medicine. The new medical school at Midlands State University has attracted young specialists to Gweru Provincial Hospital. The Medical and Dental Practitioners Council has opened a Specialist Family Practitioner Register with four members, private practitioners who specialised under the Stellenbosch University’s MMed programme. Family practitioners, on completing their MMed, will work as senior clinicians in district, mission and provincial hospitals, in City Health facilities and as supervisors of the PHC teams working in rural and urban clinics. Community-based medical education has existed in the undergraduate programme since 1987^8^ although medical officers are not trained to teach medical students who are often left to their own devices. Extending the educational system to district and mission hospitals, with family practitioners as effective educators of medical students, will be a crucial function of the family medicine programme. The result will be a more enriching education for future doctors, a better appreciation of family medicine as a speciality and more positive attitudes towards rural healthcare.
